# Protective Effects of *Cuscuta australis* Against CCl_4_-Induced Hepatic Injury in Rats: Antioxidant, Anti-Inflammatory, and In Silico Insights

**DOI:** 10.3390/ph18101524

**Published:** 2025-10-10

**Authors:** Hanen Baccari, Arij Bedoui, Anouar Feriani, Amal Bouallegue, Nihad Sahri, Sohaib Khatib, Mohamed Kharrat, Nizar Tlili, Mansour Sobeh, Moez Amri, Zouhaier Abbes

**Affiliations:** 1 Field Crop Laboratory, National Institute for Agricultural Research of Tunisia, University of Carthage, ElMenzah I, Tunis 1004, Tunisia; baccarihanen94@gmail.com (H.B.); amal.bouallegue@hotmail.fr (A.B.); kharrat.mohamed@inrat.ucar.tn (M.K.); 2 Faculty of Sciences of Gafsa, University of Gafsa, Gafsa 2012, Tunisia; 3 Laboratory of Biotechnology and Biomonitoring of the Environment and Oasis Ecosystems, Faculty of Sciences of Gafsa, University of Gafsa, Gafsa 2112, Tunisia; ferianianwer@yahoo.fr; 4 AgroBioSciences Program, College of Agriculture and Environmental Sciences, University Mohammed VI Polytechnic, Ben Guerir 43150, Morocco; nihad.sahri@um5r.ac.ma (N.S.); sohaib.khatib@um6p.ma (S.K.); mansour.sobeh@um6p.ma (M.S.); moez.amri@um6p.ma (M.A.); 5 Physio-Chemical Laboratory of Inorganic and Organic Materials, Materials Science Center, Ecole Normale Supérieure, Mohammed V University, Rabat 10001, Morocco; 6 Laboratory of Environmental Sciences and Technologies, Higher Institute of Environmental Sciences and Technologies of Borj-Cedria, PB-1003, Hammam-Lif 2050, Tunisia; nizar_fst@yahoo.fr

**Keywords:** *Cuscuta australis*, LC-MS, hepatopreventive, anti-inflammatory, oxidative stress, molecular docking

## Abstract

**Background/Objectives:** The search for new bioactive molecules increasingly extends beyond conventional medicinal plants, highlighting the importance of exploring alternative botanical sources. Parasitic plants represent a promising but underexploited reservoir of pharmacologically relevant compounds. *Cuscuta australis* (CA), a parasitic species with a history of traditional use, remains poorly characterized. This study aimed to investigate its phytochemical composition and evaluate its antioxidant, anti-inflammatory, and hepatoprotective properties. **Methods:** The phytochemical profile of CA extract was characterized by LC-MS. Antioxidant capacity was assessed using DPPH and ABTS assays. In vivo hepatoprotection was evaluated in male rats subjected to CCl_4_-induced hepatotoxicity and treated orally with CA (30 or 60 mg/kg body weight). Biochemical, lipid, oxidative stress, and histological parameters were determined. Molecular docking was conducted to predict the binding of major identified compounds against selected protein targets. **Results:** CA significantly and dose-dependently improved biochemical and histological markers. At 60 mg/kg, ALT, AST, ALP, and bilirubin were reduced by 32%, 33%, 63%, and 51%, respectively. Lipid metabolism was improved by decreased TC, TG, and LDL-C with increased HDL-C. Antioxidant defense was enhanced through elevated CAT, SOD, and GPx activities, accompanied by reduced MDA levels. TNF-α and IL-6 decreased by 48% and 53%, respectively. Histopathology confirmed hepatoprotection and reduced fibrosis. Docking studies revealed strong binding affinities (−7.07 to −19.20 kcal/mol) for several metabolites, notably quercetin glucoside, diosmetin glucoside, caffeic acid glucoside, feruloylquinic acid, and isorhamnetin glucoside, against CYP450, IL-2, TNF-α, and IL-6. **Conclusions:** These findings demonstrate that *C. australis* is a promising source of bioactive compounds with hepatoprotective, antioxidant, antihyperlipidemic, and anti-inflammatory effects, supporting its potential as a natural therapeutic agent.

## 1. Introduction

Liver diseases represent a significant global health burden, affecting millions of people worldwide and contributing to substantial morbidity, mortality, and healthcare costs [1]. Hepatic disorders, including hepatitis, fibrosis, and cirrhosis, may result from viral infections, alcohol abuse, metabolic disturbances, or exposure to environmental hepatotoxic agents [2]. Current hepatoprotective treatments, including pharmaceuticals and dietary interventions, often show limited efficacy and may cause adverse side effects [3], highlighting the need for safe, natural, and more effective therapeutic agents [4]. In this context, parasitic plants, despite their detrimental impact on crops, have attracted increasing attention due to their rich content of bioactive compounds with antioxidant, anti-inflammatory, and hepatoprotective properties [5,6].

Parasitic plants are a remarkably biodiverse group, accounting for over 4750 species, and are characterized by their distinctive nutrient uptake and survival strategies [7]. Driven by agricultural concerns, the majority of scientific research has focused primarily on a few genera, mainly *Orobanche*, *Phelipanche*, *Striga*, and *Cuscuta*, with an emphasis on their damaging effects and on methods for their management and control [8]. This focus has limited insights into other aspects of these plants, such as their chemical composition and potential therapeutic applications [9].

These plants have long been used for food, traditional medicine, and cultural practices [10,11]. Furthermore, they contain a remarkable array of bioactive molecules, including alkaloids, flavonoids, terpenes, and phenolic compounds, which may offer promising biological activities, particularly for therapeutic applications [12].

The *Convolvulaceae* family is one of the most frequently reported and studied parasitic plant families for its therapeutic properties [13,14]. Within this family, the genus *Cuscuta*, commonly known as dodder, comprises approximately 200 stem- and leaf-parasitic species [15]. Most of them depend entirely on the host for their growth and survival due to their lack of photosynthetic ability [16].

These parasites attach to the stems of their host plants via specialized organs called haustoria, which allow them to penetrate the host’s vascular tissues and absorb water and nutrients [17]. *Cuscuta australis* is a cosmopolitan annual holoparasitic plant that thrives in tropical, subtropical, and warm temperate regions, and can be found in multiple countries of the Mediterranean region [18,19,20]. Morphologically, *C. australis* is a yellow-orange, leafless shrub with thread-like stems that smothers its host plant [21].

Despite their parasitic status, recent studies have shown increasing interest in the nutraceutical and pharmacological potential of various *Cuscuta* species, highlighting their role in the modulation and prevention of several metabolic pathologies [22]. Previous results have outlined several biological properties, such as antidiabetic [23], antitumor [24], neuroprotective [22,25], and antibiofilm [26] activities.

Carbon tetrachloride (CCl_4_) is a well-known hepatotoxic agent commonly used in experimental animal models to investigate liver injury, due to its reproducible effects and well-characterized mechanisms of toxicity [27]. The liver is particularly susceptible to CCl_4_ toxicity due to its high expression of cytochrome P450 enzymes, which catalyze the bioactivation of CCl_4_ into highly reactive radicals, namely the trichloromethyl radical (•CCl_3_) and the trichloromethyl peroxyl radical (•OOCCl_3_) [28].

To our knowledge, little data is available on the chemical composition and the therapeutic potential of *C. australis.* Therefore, the present study aimed to identify its bioactive content, evaluate its in vitro antioxidant properties, and investigate its hepatoprotective effect against CCl_4_-induced hepatotoxicity in rats. Moreover, an in silico assessment was performed to support the potential anti-inflammatory and anti-fibrotic effects of *CA* compounds.

## 2. Results

### 2.1. In Vitro Antioxidant Potential and Phytocontents Dosage

Table 1 presents the results of the phytochemical content and the in vitro antioxidant activity. The data showed that CA contained high levels of polyphenols, flavonoids, and tannins. The extract also demonstrated strong antioxidant activity, as shown by two complementary tests (DPPH and ABTS). The extract’s ability to neutralize free radicals was comparable to that of the standard antioxidant, ascorbic acid.

### 2.2. Phytochemical Characterization

**Figure 1:**
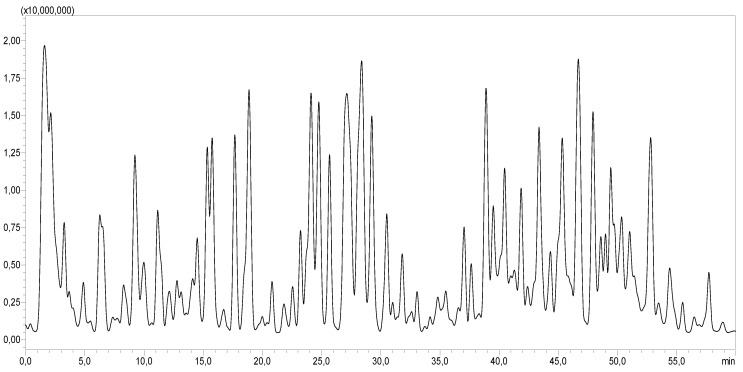
LC-MS/MS profile of *CA*.

The LC-MS/MS analysis demonstrated that CA was rich in a diverse array of bioactive molecules. As shown in Table 2 and Figure 1, a total of 51 different compounds were identified. The extract contained various organic acids, including quinic acid, malic acid, citric acid, and glucuronic acid. It also contained several phenolic acids such as gallic acid, protocatechuic acid, caffeic acid, ferulic acid, and coumaric acid. Moreover, CA was a source of flavonoids, notably quercetin, kaempferol, isorhamnetin, and diosmetin, as well as hydroxycinnamic acid derivatives like caffeoylquinic acid and tricaffeoylquinic acid.

### 2.3. In Vivo Acute Toxicity

During the observation period, no unusual behavioral or physical abnormalities were observed. Body weight, food and water intake, general behavior, and clinical signs were carefully monitored. No signs of toxicity or adverse effects were detected at the tested doses of CA. These results indicated that CA was safe and well-tolerated by the rats.

### 2.4. Effect of CA on Gravimetric Parameters

As presented in Table 3, rats treated with CCl_4_ showed a 19% decrease (*p* < 0.001) in body weight compared to the control group. This weight loss was significantly attenuated (*p* < 0.01) by the treatment with CA1 and CA2. Similar results were observed for the relative liver weight. Rats treated with CCl_4_ showed a 51% decrease (*p* < 0.001) compared to the control group. This reduction was significantly reversed by CA1 and CA2 treatments.

### 2.5. Effect of CA on Hepatic Injury Markers

**Figure 2:**
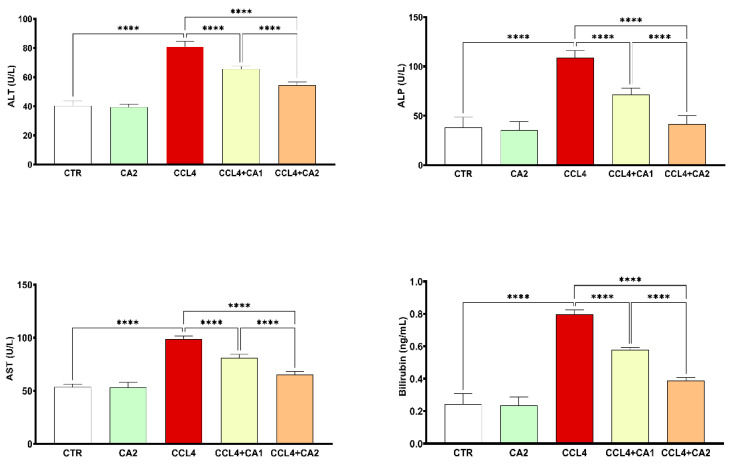
Effect of CA on hepatic injury markers. Values are given as mean ± SD for groups of six rats (n = 6) **** *p* < 0.0001: CCl_4_ vs. CTR and **** *p* < 0.0001: CCl_4_ + CA1, CCl4 + CA2 vs. CCl_4_.

Figure 2 shows the hepatic injury marker levels across the five experimental groups. CCl_4_ administration resulted in marked increases in ALT (100%), AST (85%), ALP (184%), and bilirubin (295%) levels compared to the control group. The pretreatment with CA significantly attenuated these elevations (*p* < 0.0001). Notably, in the CA2 + CCl_4_ group, the levels of ALT, AST, ALP, and bilirubin were reduced by 32%, 33%, 63%, and 51%, respectively, relative to the CCl_4_ group.

### 2.6. Effect of CA on Lipid Profile

**Figure 3:**
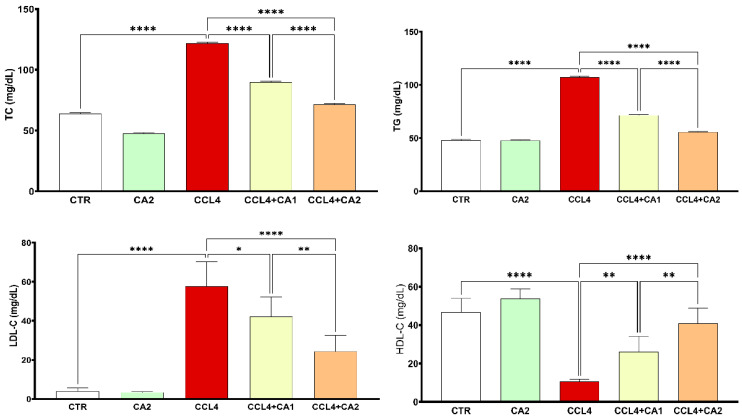
Effect of CA on lipid profile. Values are given as mean± SD for groups of six rats (n = 6) **** *p* < 0.0001: CCl_4_ vs. CTR and * *p* < 0.05; ** *p* < 0.01 and **** *p* < 0.0001: CCl_4_ + CA1, CCl_4_ + CA2 vs. CCl_4_.

The results presented in Figure 3 show that rats treated with CCl_4_ exhibited a marked increase in plasma levels of TC (90%), TG (116%), and LDL-C (136%), along with a 78% reduction in HDL-C, compared to the control group. In contrast, the pretreatment with CA significantly improved all lipid profile parameters relative to the CCl_4_ group. Specifically, TC, TG, and LDL-C levels were markedly reduced, while HDL-C levels were significantly elevated (*p* < 0.0001).

### 2.7. Effect of CA on Oxidative Stress

**Figure 4:**
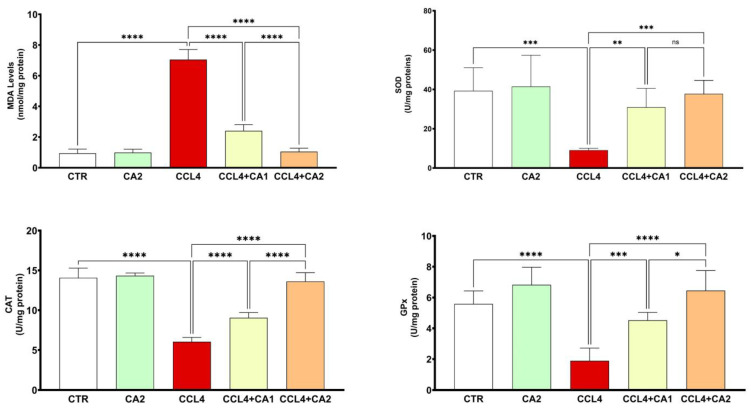
Effect of CA on pro and antioxidant enzymes. Values are given as mean ± SD for groups of six rats (*n* = 6) **** *p* < 0.0001: CCl_4_ vs. CTR, ** *p* < 0.01; *** *p* < 0.001 and **** *p* < 0.0001: CCl_4_ + CA1or CCl_4_ + CA2 vs. CCl_4_. and ns = not significant; * *p* < 0.05 and **** *p* < 0.0001: CCl_4_ + CA1vs CCl_4_ +CA2.

The data shown in Figure 4 reveal that animals exposed to CCl_4_ exhibited a marked increase in hepatic MDA levels, rising by 61% compared to the control group. However, pretreatment with CA significantly reduced lipid peroxidation by 4% relative to the CCl_4_ group, bringing MDA levels closer to those observed in untreated animals (*p* < 0.0001).

Figure 4 also highlights the beneficial effect of CA treatment on antioxidant enzyme activities in hepatic tissue. Notably, CCl_4_ administration resulted in a significant reduction in the activities of SOD (69%), CAT (57%), and GPx (66%) compared to the control group. In contrast, pretreatment with CA markedly restored the activities of these enzymes relative to the CCl_4_ group (*p* < 0.001).

### 2.8. Effect of CA on Pro-Inflammatory Cytokine Levels

**Figure 5:**
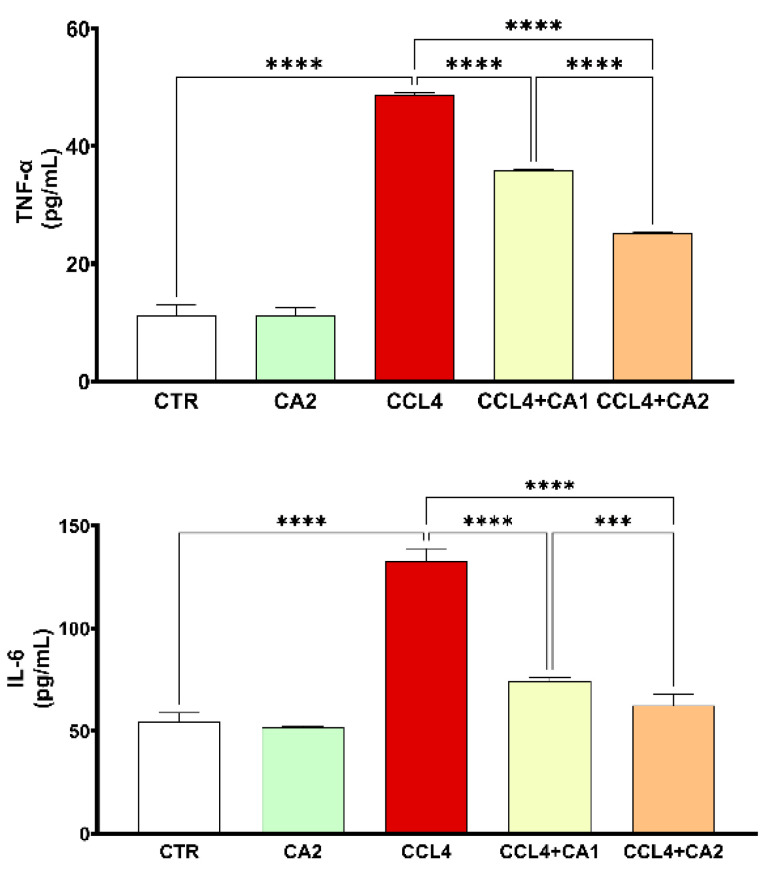
Effect of CA on the levels of pro-inflammatory cytokines. Values are given as mean ± SD for groups of six rats (n = 6) **** *p* < 0.0001: CCl_4_ vs. CTR and **** *p* < 0.0001: CCl_4_ + CA1, CCl_4_ + CA2 vs. CCl_4_. *** *p* < 0.001: CCl_4_ + CA1vs CCl_4_ +CA2.

Both TNF-α and IL-6 are key pro-inflammatory cytokines, and their measurement confirms the presence of liver inflammation. As shown in Figure 5, CCl_4_ administration significantly increased TNF-α and IL-6 levels by 33% and 150%, respectively, compared to the control group. However, treatment with CA1 or CA2 significantly reduced these elevated levels (*p* < 0.0001). In the CCl_4_ + CA1 group, TNF-α and IL-6 levels decreased by 26% and 43%, respectively, while in the CCl_4_ + CA2 group, greater reductions were observed, 48% for TNF-α and 53% for IL-6.

### 2.9. Histological Study

**Figure 6:**
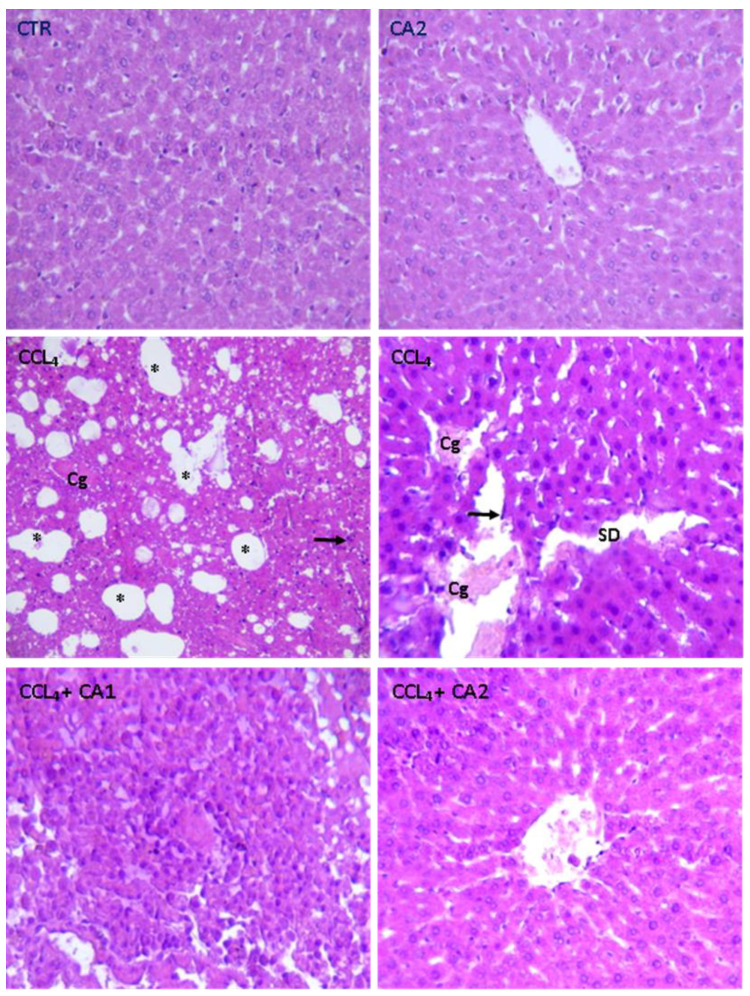
Effect of CA on the structure of liver tissues stained with Hematoxylin and Eosin (GX200) in all experimental groups of rats. Cg: Congestion of the centrilobular vein; Arrow black: Infiltration Leucocyte; SD: Sinusoidal dilation; *: Foci of lipid.

Figure 6 and Table 4 present the histological findings from H&E-stained liver sections of the control and treated groups. In both the control and CA-treated groups, hepatic architecture was preserved, with polyhedral hepatocytes arranged radially around the central vein and separated by intact sinusoids. However, the CCl_4_ group showed pronounced pathological alterations, including sinusoidal dilation, severe congestion of the centrilobular vein, and infiltration of inflammatory cells. Pretreatment with CA at both doses significantly attenuated most of the CCl_4_-induced alterations.

Sirius Red staining was employed to visualize collagen fibers in liver tissue (Figure S1). The CCl_4_-treated group showed extensive red-stained regions, indicating abnormal collagen buildup consistent with fibrotic lesions that distort normal hepatic architecture. Semi-quantitative morphometric analysis confirmed a significant increase in collagen-positive areas compared to controls (*p* < 0.0001). Treatment with *Cuscuta australis* significantly decreased collagen deposition, helping to restore a more intact hepatic structure. Overall, the Sirius Red results highlight fibrotic lesions rather than necrosis or inflammation, aligning with the typical pathology of CCl_4_-induced chronic liver injury.

### 2.10. Molecular Docking

Several identified compounds from the extract were selected and subjected to molecular docking with CYP450, IL-2, IL-6, and TNF-α. Some of these compounds demonstrated strong binding affinities, as summarized in Table 5.

For CYP450, several phytoconstituents exhibited higher affinities than the reference compound silymarin (−16.77 kcal/mol) and the co-crystalized ligand (−6.29 kcal/mol). The most favorable docking scores were recorded for quercetin glucoside (−19.20 kcal/mol), feruloylquinic acid (−18.00 kcal/mol), diosmetin glucoside (−17.85 kcal/mol), coumaric acid glucoside (−17.75 kcal/mol), and isorhamnetin glucoside (−17.44 kcal/mol). These ligands engaged in multiple hydrogen bonds and ionic interactions with key catalytic residues such as Arg 100, Arg 126, Arg 435, and Trp 122, indicating potential inhibitory effects that may surpass those of silymarin.

With IL-2, several compounds showed remarkable affinities, often exceeding the standard (Silymarin, −10.30 kcal/mol). Notably, quercetin glucoside (−11.09 kcal/mol), kaempferol (−12.31 kcal/mol), gallic acid (−12.25 kcal/mol), diosmetin glucoside (−11.03 kcal/mol), caffeic acid (−11.03 kcal/mol), and isorhamnetin glucoside (−10.03 kcal/mol) displayed appreciable interactions. These results suggest that glycosylated flavonoids and phenolic acids could act as effective modulators of IL-2 activity through stable hydrogen bonding with residues including Lys 64, Asn 88, and Glu 116.

Docking against IL-6 identified quercetin glucoside (−16.47 kcal/mol) as the strongest binder, markedly outperforming silymarin (−13.43 kcal/mol). Other compounds with notable affinities included caffeoylquinic acid (−14.39 kcal/mol), isorhamnetin glucoside (−14.13 kcal/mol), diosmetin (−13.02 kcal/mol), feruloylquinic acid (−13.17 kcal/mol), and quercetin (−13.25 kcal/mol). These findings highlight the enhanced interaction potential of glycosylated flavonoids and esterified phenolic acids with IL-6, suggesting their contribution to the extract’s anti-inflammatory activity.

**Figure 7:**
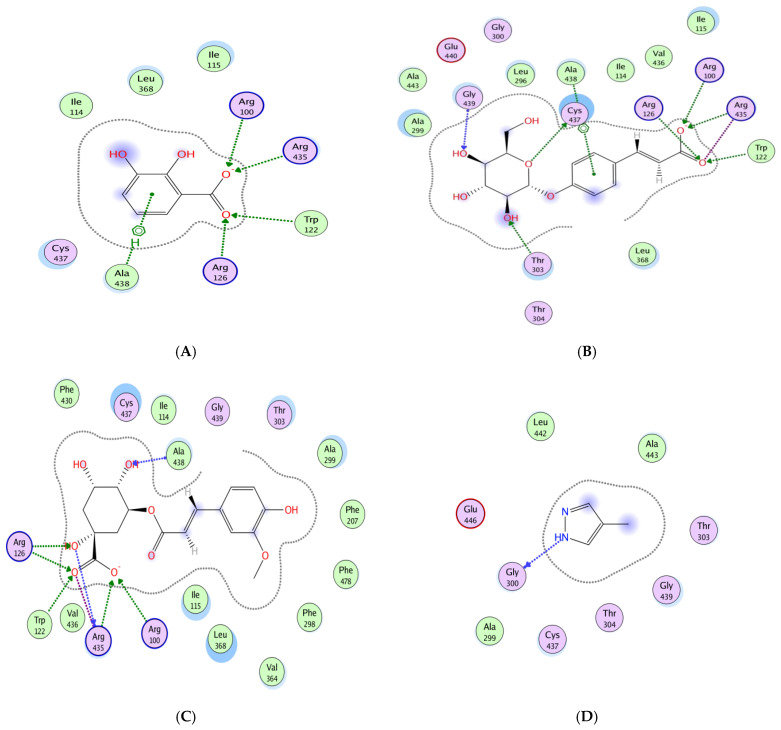
Molecular docking of dihydroxybenzoic acid (**A**), caffeic acid glucoside (**B**), feruloylquinic acid (**C**), and co-crystallized ligand 4-methylpyrazole (**D**), with CYP450 (3E4E) active site.

The most striking binding profiles were observed with TNF-α. Several compounds surpassed silymarin (−8.35 kcal/mol), including caffeic acid glucoside (−13.11 kcal/mol), quercetin glucoside (−11.87 kcal/mol), isorhamnetin glucoside (−10.69 kcal/mol), and diosmetin glucoside (−10.61 kcal/mol). These strong affinities were supported by hydrogen bond formation with residues such as Lys 87 and Leu 233, indicating a potential inhibitory effect on TNF-α signaling. Overall, quercetin glucoside, diosmetin glucoside, caffeic acid glucoside, and isorhamnetin glucoside consistently demonstrated high binding affinities across all four targets (−10.30 to −19.20 kcal/mol) and engaged in multiple stabilizing interactions. This broad-spectrum binding profile underscores their likely role as principal contributors to the biological activity of *C. australis* extract (Table S1, Figure 7).

## 3. Discussion

This study aimed to provide new insights into the bioactive potential of the parasitic plant *C. australis* and to evaluate the therapeutic efficacy of its hydroethanolic extract (CA) against CCl_4_-induced hepatic injury in rats.

LC-MS/MS analysis of CA revealed 51 bioactive compounds, including phenolic acids and flavonoids known for their antioxidant, anti-inflammatory, and hepatoprotective effects, such as protocatechuic acid [29], caffeic acid [30], ferulic acid [31], quercetin [32], kaempferol [33], and diosmetin [34]. In addition to these metabolites, the high degree of glycosylation, particularly in flavonoids and hydroxycinnamic acid derivatives, may play a crucial role in enhancing compound solubility and bioavailability, as reported by Dahiya et al. [35]. The phytochemical profile of *C. australis* supported the idea that some parasitic plants can serve as valuable sources of bioactive molecules for nutraceutical and therapeutic applications. Indeed, recent studies have highlighted the richness of parasitic plants in health-promoting compounds [11,12,27].

The in vitro assays demonstrated that the molecules identified in CA possess significant antioxidant capacity. Indeed, recent studies using various antioxidant assays have highlighted the strong antioxidant potential of several *Cuscuta* species, notably *C. campestris* and *C. reflexa* [24,36].

The findings of the present study revealed that exposure to CCl_4_ significantly decreased both body weight and relative liver weight compared to the control rats, clearly illustrating the systemic and hepatotoxic effects of CCl_4_ [37].

Lipid accumulation in the bloodstream is a major risk factor for hepatic inflammation, particularly in steatotic liver diseases [38]. In the present study, we evaluated the plasma lipid profile, as disturbances in lipid metabolism are commonly associated with liver injury and hepatotoxicity. Assessing lipid parameters alongside biochemical markers, oxidative stress indicators, and histological analyses provides a comprehensive evaluation of the hepatoprotective potential of *C. australis* (CA). The ability of CA to normalize the plasma lipid profile, evidenced by reductions in total cholesterol (TC), triglycerides (TG), and LDL-C levels, may be attributed to the inhibition of pancreatic lipase and HMG-CoA reductase, the key enzyme involved in cholesterol synthesis, as reported in previous studies [39,40]. Furthermore, the observed increase in HDL-C levels might result from modulation of the SR-BI receptor, enhancing reverse cholesterol transport, consistent with earlier findings [41,42]. These results suggested that CA not only protected liver structure and function but also helped maintain normal lipid metabolism, highlighting its broader hepatopreventive effects.

The significant reduction in elevated hepatic injury biomarkers AST, ALT, ALP, and bilirubin, following CA administration, suggested the presence of bioactive compounds capable of protecting the liver against CCl_4_-induced damage. This hepatoprotective effect might be attributed to the preservation of hepatocyte membrane integrity and the enhancement of overall liver function, as previously reported [43].

Similar hepatoprotective effects have been reported for ethanolic extracts of *C. australis* stem and seed in acetaminophen-induced liver injury [44], *C. arvensis* in APAP models [45], and *C. campestris* whole-plant extract in CCl_4_ models [46]. Additionally, a broader review on the hepatoprotective properties of *C. reflexa* further supports these findings [47].

The observed reduction in MDA levels in the CA + CCl_4_ groups was likely attributed to the antioxidant capacity of CA, mediated by its bioactive metabolites, several of which are well-documented for their potent radical-scavenging properties against reactive oxygen species (ROS) [48]. Moreover, CA treatment enhanced the activities of hepatic antioxidant enzymes (SOD, CAT, and GPx). These findings suggested that *CA* might stimulate endogenous antioxidant defenses and provide protection against oxidative stress, a mechanism closely associated with hepatic diseases [49].

Our results were consistent with those of Folarin et al. [44] and Zhang et al. [50], who reported the antioxidant and hepatoprotective activities of the same species using stem and seed extracts. While these studies demonstrated the pharmacological potential of selected plant parts, they did not include a detailed phytochemical characterization. In contrast, the present work made a novel contribution by evaluating the entire plant and characterizing its bioactive constituents. This comprehensive approach seemed to be important, as the combined phytochemical profile of the whole plant might exert synergistic effects and offer broader biological activity. By integrating both whole-plant assessment and phytochemical profiling, the current study not only complemented previous findings but also expanded the scientific understanding of this species and its potential applications in hepatoprotection.

By reducing pro-inflammatory cytokine levels, CA demonstrated its potential to counteract oxidative stress, which acted as the initial trigger of the inflammatory response. In the context of CCl_4_-induced liver injury, ROS not only caused direct hepatocellular damage but also initiated inflammatory signaling by activating Kupffer cells, which in turn release TNF-α.

A study by Sato et al., [51] demonstrated that CD11b^+^ Kupffer cells produce TNF-α and FasL, playing a pivotal role in CCl_4_-induced acute hepatic injury. The activation of these cells led to the release of pro-inflammatory cytokines, including TNF-α, which further aggravated liver damage. This finding underscored the critical involvement of Kupffer cells in the inflammatory processes associated with CCl_4_-induced liver injury.

Additionally, a review by [52] highlighted the diverse functions of macrophages, including Kupffer cells, in experimental liver injury. The authors emphasized that macrophages play a significant role in liver repair and regeneration following injury, suggesting that targeting these cells could represent a therapeutic strategy for liver diseases.

This cytokine subsequently stimulates the secretion of IL-1β and IL-6, thereby amplifying inflammation and leading to hepatic necrosis and biochemical disturbances [53].

Hematoxylin-eosin (H&E) staining revealed that CA pretreatment significantly attenuated CCl_4_-induced alterations, such as centrilobular necrosis and inflammatory cell infiltration in the hepatic tissues of rats. Remarkably, these histological improvements were consistent with the biochemical findings, further supporting the hepatoprotective potential of CA. Notably, other parasitic plants demonstrated similar protective effects in previous studies [27,54]. Hepatic fibrosis is well known as a major consequence of liver damage caused by toxic agents such as CCl_4_ and is characterized by excessive deposition of collagen proteins in hepatic tissue [55]. Since H&E staining does not allow clear visualization of fibrosis, special histological stains such as Sirius Red or Masson’s Trichrome are typically used, as proposed previously [42]. In the present study, Sirius Red staining confirmed that CCl_4_ exposure induced significant liver fibrosis, as evidenced by increased collagen accumulation. These observations were consistent with previous reports [55]. The fibrotic process is attributed to the activation of Kupffer cells and hepatic stellate cells (HSCs), which transdifferentiate into myofibroblasts and subsequently produce extracellular matrix components, including collagen [56].

Molecular docking analysis was carried out to explore the interaction between CA-derived compounds and proteins involved in the inflammatory response and hepatic fibrosis. The results revealed strong and stable binding affinities with four key molecular targets: CYP450, IL-2, TNF-α, and IL-6, suggesting a potential inhibitory effect. These proteins were selected based on their established roles in hepatic pathophysiology, including CYP450 in CCl_4_-induced toxicity [57] while IL-6, IL-2, and TNF-α are recognized hallmarks of the inflammatory response [58]. In the present study, IL-6 and TNF-α were measured in vivo as key inflammatory markers directly involved in CCl_4_-induced hepatotoxicity, whereas IL-2 was included in the docking analysis to explore additional immune-modulatory pathways since it plays a central role in immune modulation and T-cell activation, which are also closely linked to inflammation and liver injury [59]. The inclusion of IL-2 allowed us to broaden the mechanistic insight into the potential anti-inflammatory and hepatoprotective effects of *C. australis*, thereby complementing the experimental findings. On the other hand, the RMSD values were all below 3 Å, securing that the implemented docking method was reliable and reproducible.

Among the docked molecules, the majority of compounds namely quercetin, caffeic acid glucoside, caffeoylquinic acid, ferulic acid glucoside, feruloylquinic acid, diosmetin glucoside, quercetin glucoside, isorhamnetin glucoside, and caffeoyl coumaroylquinic acid exhibited remarkable binding affinities for the four selected proteins.

The docking results of quercetin glucoside demonstrated strong affinity and multiple stabilizing interactions with pro-inflammatory cytokines, which may support its anti-inflammatory potential. In line with this, this flavonoid glycoside, is recognized for its antioxidant, anti-inflammatory and hepatoprotective properties, as reported in several recent studies [60,61]. Structurally, this compound possesses features that enhance its antioxidant capacity. Notably, the occurrence of the hydroxyl groups helps donate hydrogen atoms and supports radical stabilization, making it an effective antioxidant. Compared to quercetin itself, its glycate derivative offered higher cellular defense against oxidative stress, thanks to its increased solubility, which facilitated better uptake and distribution within the cell [62].

Taken together, these findings suggest that *C. australis* extract (CA) exerts antioxidant, anti-inflammatory, antifibrotic, and hepatoprotective effects, supporting its potential as a natural complementary strategy for liver protection. The translational relevance of this work lies in the possibility of developing CA-based formulations or nutraceuticals to mitigate liver damage associated with oxidative stress and inflammation. Nevertheless, several limitations should be acknowledged. First, the antioxidant activity results may vary with dilution volume and reaction conditions, which can affect reproducibility and comparison between studies. Therefore, this antioxidant activity assessment should be regarded as a mere preliminary investigation of the therapeutic potential rather than a definitive evaluation. Second, the study was conducted in an animal model, and the results cannot be directly extrapolated to humans without further preclinical and clinical validation. Third, only two doses were tested, and a more comprehensive dose–response assessment is required to fully establish the therapeutic window. Furthermore, the pharmacokinetics, bioavailability and potential toxicity of the identified phytoconstituents remain to be clarified. Finally, while molecular docking provided valuable mechanistic insights, these predicted interactions require experimental validation. Future studies should therefore focus on pharmacokinetic investigations, molecular target confirmation, and well-designed clinical trials to confirm the hepatoprotective efficacy of *C. australis* in humans.

## 4. Materials and Methods

### 4.1. Chemicals and Standards

The standard compounds, CCl_4_, and biochemical assay kits were supplied by Sigma-Aldrich (St. Louis, MO, USA). For mass spectrometry analyses, chemicals of MS quality were used, ensuring a high level of purity suitable for such analyses. LC-grade acetic acid and acetonitrile were obtained from Fluka (Buchs, SG, Switzerland) and Thermo Fisher (Waltham, MA, USA), respectively.

### 4.2. Plant Harvesting and Extraction

At maturity, the whole plants of *CA* were collected in April 2022 from the Oued Beja region, located in northwestern Tunisia (36°43′01″ N; 9°13′18″ E). The harvested plants were air-dried in a shaded and aerated room. The dried samples were ground, and 350 g of the dried plant material were stored in a closed glass container at −20 °C until use. For the extraction, the method described by Najahi et al., [63] was followed. 100 g of the powder was extracted using an ethanol-water solution (80:20, *v*/*v*), in an ultrasonic bath (100 W, 40 kHz; Ultrasonic, J.P. Selecta, Barcelona, Spain). The mixture was stirred for 72 h, then centrifuged at 4000 rpm for 15 min at 4 °C. After that, the resulting supernatant was filtered using 0.45 μm nylon syringe filters (SinerLab Group, Madrid, Spain). The filtered solution was concentrated under reduced pressure using a rotary evaporator. The obtained residues were fully dried to ensure complete ethanol removal prior to biological assays. Finally, the obtained extract (CA) was stored in a sealed glass container at 4 °C.

### 4.3. In Vitro Assays

The phytochemical content and the antioxidant properties of CA were estimated through in vitro spectrophotometric methods. The total phenolic content (TPC) was determined using the Folin–Ciocalteu method [64]. The absorbance was measured at 760 nm. The results were expressed as milligrams of gallic acid equivalents per gram of dry weight (mg GAE/g DW). The total flavonoid content (TFC) was quantified using a colorimetric assay, involving the mixture of 5 μL of 5% NaNO_2_, and 150 μL of 10% AlCl_3_ with the CA sample [65]. The absorbance was measured at 510 nm. The results were expressed as milligrams of quercetin equivalents per gram of dry weight (mg QE/g DW). The evaluation of the total tannin content (TTC) [66] was performed by mixing 20 µL of the sample with 600 µL of a 4% vanillin solution in methanol. After 15 min, 300 µL of concentrated HCl was added. Then, the absorbance was read at 500 nm. The results were expressed as milligrams of catechin equivalents per gram dry weight (mg CE/g DW).

The antioxidant capacity of CA was assessed using DPPH and ABTS radical scavenging assays [67]. The absorbances were measured at 517 nm and 734 nm, respectively. The radical’s inhibition was calculated using the following formula:

Radicals’ inhibition (%) = (1 − A1/A0) × 100,

where A0: the absorbance of the control; A1: The absorbance of the tested sample.

Vitamin C was used as a positive control.

To ensure the accuracy of antioxidant activity measurements, potential self-bleaching of free radicals was carefully monitored, and appropriate blanks were included to correct for spontaneous radical decay.

### 4.4. HPLC-PDA-MS/MS Analyses

A HPLC-ESI-MS/MS system was used in order to carry out the CA composition according to the protocol described by Bedoui et al. [67]. This system included a Shimadzu entity connected to an MS 8050 mass spectrometer equipped with an electrospray ionization source. Chromatographic separation was performed on a Zorbax Eclipse XDB-C18 column (4.6 × 150 mm, 3.5 µm; Agilent Technologies, Santa Clara, CA, USA), maintained at 30 °C. Water and acetonitrile were used as the mobile phases, both mixed with 0.1% formic acid, at a flow rate of 1 mL/min. The gradient program increased from 5% acetonitrile to 30% after 45 min, and then to 90% over the following 15 min. The collision energy was set to −35 eV, and the mass spectrometer was operated in a scanning mode from 100 to 1500 m/z. The sample was injected using the SIL-40C XS autosampler (Shimadzu Scientific Instruments, Columbia, MD, USA).

### 4.5. In Vivo Assays

#### 4.5.1. Animals

The study was conducted on male Wistar rats weighing approximately 200 ± 30 g. The rats were obtained from the Pasteur Institute of Tunis. The rats were housed under controlled conditions, in appropriate laboratory cages at the Faculty of Sciences of Gafsa, Tunisia. They were provided with a standard chow diet and had free access to water.

#### 4.5.2. Acute Toxicity Assessment

To assess any possible toxic effect of CA, an acute toxicity test was performed. Thirty rats were divided into six groups (n = 6). The first group was the control group that did not receive any treatment, while the other groups each received a gradient dose of CA (10, 20, 30, 40, 60 mg/kg body weight). All rats were carefully monitored for clinical signs of toxicity, body weight changes, food and water intake, and general behavior during the first 24 h and throughout the three-week follow-up period.

#### 4.5.3. Experimental Design

In order to test the potential hepatoprotective effects of *C. australis* (CA), the treatments were conducted according to the method described by Taamalli et al. [68]. After 15 days of adaptation and observation period, male rats were randomly divided into five groups (n = 6) using a simple randomization procedure.

The assay was conducted for six weeks, with the treatment protocols for each group as follows:

Group I (Control, CTR): Rats received 1 mL of corn oil by gavage;

Group II (CA2): Rats received 1 mL of *C. australis* (60 mg/kg b.w.) dissolved in corn oil by gavage;

Group III (CCl_4_): Rats were intraperitoneally injected with CCl_4_ at 2 mL/kg b.w.;

Group IV (CA1 + CCl_4_): Rats received daily 1 mL of *C. australis* at 30 mg/kg b.w. (referred to us CA1) dissolved in corn oil by gavage. After 7 days of pretreatment, they were intraperitoneally injected with CCl_4_ dissolved in corn oil;

Group V (CA2 + CCl_4_): Rats received daily 1 mL of *C. australis* at 60 mg/kg b.w. (referred to us CA2) dissolved in corn oil by gavage. After 7 days of pretreatment, they were intraperitoneally injected with CCl_4_ dissolved in corn oil.

Pretreatment with CA1 or CA2 was administered daily for 7 days prior to the first CCl_4_ injection and continued throughout the study period.

At the end of the treatments, rats were sacrificed by cervical decapitation. The blood and the liver were collected. The plasma was separated by centrifugation at 2000× *g* for 15 min and then stored together with parts of the liver at −20 °C for biochemical and molecular analysis. The remaining liver tissues were fixed in 10% formalin for histological examination.

All biochemical, histological, and data analyses were performed by researchers blinded to the group allocations to minimize bias.

#### 4.5.4. Lipid Profile

Kits from BIOMAGHREB (La Goulette, Tunisia) were used to analyze the lipid profile by measuring plasma levels of LDL-C, HDL-C, total cholesterol (TC), and triglycerides (TG), according to the manufacturer’s guidelines.

#### 4.5.5. Hepatic Functional Enzymes

The activities of hepatic enzymes, including aspartate aminotransferase (AST), alanine aminotransferase (ALT), alkaline phosphatase (ALP), and plasma bilirubin, were measured spectrophotometrically according to the manufacturer’s instructions. The levels of the pro-inflammatory cytokines IL-6 and TNF-α in the blood samples were measured using the ELISA assay, with diagnostic kits from BIOMAGHREB (La Goulette, Tunisia).

#### 4.5.6. Oxidative Stress Markers

Liver tissue was placed on ice, washed with normal saline solution, and then homogenized in potassium phosphate buffer (0.1 M, pH 7.4). The obtained homogenate was centrifuged at 12,000 rpm for 15 min at 4 °C. The supernatant was used to assess malondialdehyde (MDA) levels [69] and to measure the activities of superoxide dismutase (SOD) [70], catalase [71], and glutathione peroxidase (GPx) [72]. The protein content of liver was measured using the Bradford method, with bovine serum albumin as the standard.

#### 4.5.7. Histopathological Examinations

Harvested liver tissues were collected, rinsed with saline solution, and fixed for 24 h in 10% buffered formalin. The samples were then dehydrated, cleared in xylene, and embedded in paraffin. Sections of 4–6 μm thickness were prepared using a rotary microtome and stained with hematoxylin-eosin (H-E) for histopathological examination. Tissue images were captured at ×200 magnification using a light microscope to analyze the morphological structure of the liver in each experimental group. To measure liver fibrosis, tissue samples were stained with Sirius red to highlight collagen in red. For each sample, ten random areas were analyzed under the microscope. The red-stained area was measured using ImageJ 1.49v NIH software, and the results were expressed as the percentage of fibrosis in each area [55].

### 4.6. Molecular Docking

#### 4.6.1. Phytoligands Preparation

The three-dimensional structures of the major metabolites (<500 Da) identified by LC-MS/MS were downloaded from the PubChem database (SDF format) and compiled into a single ligand dataset. Prior to docking, all ligands were prepared in MOE 2022.02 (Chemical Computing Group, Montreal, QC, Canada). This process involved optimizing the structures to correct formal charges of strong acids and bases, adjusting bond lengths, and removing any structural aberrations. To ensure chemical plausibility, phytoligands were converted to their most likely tautomeric states using the Protonate3D and Protomers tools. Partial atomic charges were then assigned, and the structures were energy-minimized with the MMFF94x force field until convergence at a gradient of 0.0001 kcal·mol^−1^·Å^−1^, yielding stable geometries suitable for reliable docking analysis.

#### 4.6.2. Targets Preparation

The crystal structures of four proteins involved in hepatic injury and inflammation, namely CYP450 (3E4E), IL-2 (1M47), IL-6 (1ALU), and TNF-α (7JRA) were retrieved from the Protein Data Bank (www.rcsb.org accessed on 25 June 2025). Each structure was then processed in MOE using the QuickPrep panel to ensure suitability for docking studies. This preparation included protonating ionizable residues at physiological pH, adding missing hydrogen atoms, correcting structural irregularities, and removing unbound crystallographic water molecules that could interfere with binding-site accessibility. Finally, the structures were energy-minimized with the Amber10:EHT force field to relieve steric clashes and refine hydrogen-bond networks, resulting in stable and well-optimized protein conformations.

#### 4.6.3. Molecular Docking

The docking simulations were performed using the Triangle Matcher method, with initial poses scored by the London dG function. The best poses were then refined to allow flexibility of the ligand and nearby residues, and rescored using the GBVI/WSA dG function, which considers van der Waals forces, solvation, and electrostatics. To check the reliability of the method and avoid bias, we re-docked the co-crystallized ligands into their proteins, which gave RMSD values below 3 Å, and compared the test compounds with reference ligands. This protocol follows the guidelines for docking studies described in [73].

### 4.7. Statistics

The data are presented as means ± SD, and differences between groups were assessed using Tukey’s post hoc test. All data were subjected to one-way analysis of variance (ANOVA) with a significance level of *p* < 0.05. Statistical analysis was performed using GraphPad Prism (GraphPad Prism 10.2.0, San Diego, CA, USA).

## 5. Conclusions

This study demonstrated the hepatoprotective potential of bioactive compounds derived from *Cuscuta australis* (CA), a parasitic plant. LC-MS/MS analysis identified a rich array of bioactive molecules, including several phenolic acids and flavonoids known for their antioxidant, anti-inflammatory, and lipid-regulating properties. In vivo investigations indicated that CA successfully mitigated the progression of severe hepatic disorders, such as cirrhosis and hepatocellular carcinoma, by alleviating both physiological and structural liver damage. Furthermore, molecular docking analysis suggested that key biomolecules of *CA* may interact with targets related to inflammation, fibrosis, and hepatoprotection, particularly in the context of CCl_4_-induced hepatotoxicity. These findings highlight *C. australis* as a promising natural source of phenolic compounds with potential applications in both nutraceutical and pharmaceutical fields.

## Figures and Tables

**Table 1:** Phenolic contents and antioxidant activities of *CA*.

	TPC *	TFC **	TTC ***	DPPH ****	ABTS ****
CA	16.89 ± 2.8	10.32 ± 1.5	0.75 ± 0.1	0.58 ± 0.14	0.62 ± 0.05
Ascorbic acid	-	-	-	0.63 ± 0.07	0.73 ± 0.02

*: mg GAE/g DW; **: mg QE/g DW; ***: mg CE/g DW; ****: IC_50_ mg/mL: The values are expressed as mean ± SD (n = 3).

**Table 2:** Annotated compounds from *CA* using LC-MS/MS.

Rt (min)	[M−H]−	MS/MS	Proposed Compounds
1.46	191	108	Quinic acid
1.56	133	133	Malic acid
1.62	191	111	Citric acid
1.68	193	113	Glucuronic acid
3.60	315	108, 153	Protocatechuic acid
5.19	169		Gallic acid
6.13	153	108	Dihydroxybenzoic acid
6.49	499	163, 191	Coumaroylquinic acid glucoside
7.60	341	135, 179	Caffeic acid glucoside
7.63	325	119, 163	Coumaric acid glucoside
8.11	515	179, 191	Caffeoylquinic acid glucoside
8.89	503	135, 179	Caffeic acid diglucoside
9.79	337	163, 191	Coumaroylquinic acid
10.48	355	193	Ferulic acid glucoside
11.06	503	135, 179	Caffeic acid diglucoside
11.08	353	179, 191	Caffeoylquinic acid
11.36	499	163, 191	Caffeoyl coumaroylquinic acid
12.98	179	135	Caffeic acid
13.46	771	301, 447, 609	Quercetin diglucoside rhamnoside
15.29	337	163, 191	Coumaroylquinic acid
15.56	639	301, 315	Isorhamnetin diglucoside
17.31	625	301, 463	Quercetin diglucoside
17.57	367	191, 193	Feruloylquinic acid
18.90	337	163, 191	Coumaroylquinic acid
19.41	163	119	Coumaric acid
20.55	917	285, 593, 755	Kaempferol triglucoside rhamnoside
21.15	785	285, 623, 785	Kaempferol glucuronide diglucoside
21.33	933	463, 609, 771	Quercetin triglucoside rhamnoside
23.22	609	301	Quercetin glucoside rhamnoside
23.62	933	285, 447, 771	Kaempferol tetraglucoside
23.89	677	173, 179	Tricaffeoylquinic acid
24.04	463	301	Quercetin glucoside
24.28	917	285, 593, 755	Kaempferol triglucoside rhamnoside
24.40	947	285, 447, 623	Kaempferol glucuronide triglucoside
25.51	771	301, 447, 609	Quercetin diglucoside rhamnoside
25.66	623	315, 461	Isorhamnetin glucoside rhamnoside
27.17	593	285	Kaempferol glucoside rhamnoside
27.43	623	315	Isorhamnetin glucoside rhamnoside
27.83	801	301, 463, 639	Quercetin feruloyl glucosyl glucoside
29.21	477	315	Isorhamnetin glucoside
30.56	755	285	Kaempferol diglucoside rhamnoside
31.76	665	315, 461	Isorhamnetin acetyl glucoside rhamnoside
37.77	639	271, 301	Quercetin feruloyl glucoside
39.77	301	151, 179	Quercetin
39.07	609	285, 447	Kaempferol caffeoyl glucoside
39.64	609	301	Quercetin coumaroyl glucoside
40.45	593	285	Kaempferol coumaroyl glucoside
42.58	445	284, 299	Diosmetin glucoside
43.24	593	285	Kaempferol coumaroyl glucoside
44.26	623	285	Kaempferol feruloyl glucoside
46.61	285	151, 255	Kaempferol
48.03	315	301	Isorhamnetin
52.77	299	271, 299	Diosmetin

**Table 3:** Effect of CA on gravimetric parameters.

	CTR	CA2	CCl_4_	CCl_4_ + CA1	CCl_4_ + CA2
Body weight (g)	231.3 ± 5.1	230.3 ± 5.6	186.6 ± 5.4 ***	214.3 ± 3.8 **	213.1 ± 4.2 **
Relative weight of liver (g/100 g)	3.4 ± 0.5	3.6 ± 0.2	1.69 ± 0.5 ***	2.91 ± 0.3 **	3.41 ± 0.44 ***

Values are given as mean ± SD for groups of six rats (n = 6) *** *p* < 0.001: CCl_4_ vs. CTR and ** *p* < 0.01; *** *p* < 0.001: CCl_4_ + CA1, CCl_4_ + CA2 vs. CCl_4._

**Table 4:** Effect of CA on histopathological structure.

	CTR	CA2	CCl_4_	CCl_4_ + CA1	CCl_4_ + CA2
Normal hepatocytes	+	+	-	-	+
Intact sinusoids	+	+	-	-	+
Sinusoidal dilation	-	-	++	+	-
Congestion of the centrilobular vein	-	-	++	+	-
Infiltration Leucocyte	-	-	++	-	-
Foci of lipid	-	-	++	+	-

-Absence, + moderate, ++ severe.

**Table 5:** Docking scores.

Compounds	Docking Score (Kcal/moL)			
	CYP450 (3E4E)	IL-2 (1M47)	TNF-α (7JRA)	IL-6 (1ALU)
Malic acid	−12.91	−9.70	−8.09	−11.22
Dihydroxybenzoic acid	−10.73	−11.56	−10.52	−9.75
Coumaric acid	−13.04	−7.80	−6.41	−9.50
Gallic acid	−12.06	−12.25	-	−13.63
Caffeic acid	−13.37	−11.03	−8.62	−10.86
Quinic acid	−12.80	−7.07	−10.08	−10.11
Citric acid	−14.74	−10.94	−10.27	−11.46
Glucuronic acid	−14.53	−7.65	−11.92	−10.47
Kaempferol	−11.34	−12.31	−8.51	−10.95
Diosmetin	−12.45	−10.77	-	−13.02
Quercetin	−14.09	−10.95	−9.48	−13.25
Protocatechuic acid	−10.83	−10.10	−8.90	−8.91
Isorhamnetin	−12.52	−10.31	-	−11.52
Coumaric acid glucoside	−17.75	−8.08	−9.11	−10.88
Coumaroylquinic acid	−16.47	−9.65	−10.26	−11.21
Caffeic acid glucoside	−17.13	−8.52	−13.11	−12.25
Caffeoylquinic acid	−15.20	−9.52	−10.50	−14.39
Ferulic acid glucoside	−13.90	−7.26	−7.95	−10.14
Feruloylquinic acid	−18.00	−9.86	−7.66	−13.17
Diosmetin glucoside	−17.85	−11.03	−10.61	−10.30
Quercetin glucoside	−19.20	−11.09	−11.87	−16.47
Isorhamnetin glucoside	−17.44	−10.03	−10.69	−14.13
Silymarin^®^	−16.77	−10.30	−8.35	−13.43
Co-crystallized ligand (4-Methylpyrazole)	−6.29			

## Data Availability

Data is contained within the article and the Supplementary Materials.

## Supplementary Materials

The following supporting information can be downloaded at https://www.mdpi.com/article/10.3390/ph18101524/s1: Table S1: Molecular docking results and interaction details of the main phenolic compounds with CYP450 (3E4E), IL-2 (1M47), TNF-α (7JRA), and IL-6 (1ALU) targets, including the interacting residues and RMSD values; Figure S1: Effect of Cuscuta australis extract on liver fibrosis stained with Sirius red (GX200) in all experimental rat groups.
