# Supplementary material for: Protective Effects of Cuscuta australis Against CCl4-Induced Hepatic Injury in Rats: Antioxidant, Anti-Inflammatory, and In Silico Insights
Source: Pharmaceuticals (Basel). 2025 Oct 10;18(10):1524. doi: 10.3390/ph18101524 (PMC12566785; doi:10.3390/ph18101524)
Supplement: Supplementary file 1 [file pharmaceuticals-18-01524-s001.zip › pharmaceuticals-3827304-supplementary.pdf]

Table_S1. Molecular docking results and interaction details.

Compounds	CYP450 (3E4E)		IL-2 (1M47)		TNF-α (7JRA)		IL-6 (1ALU)	
	Interactions	RMSD	Interactions	RMSD	Interactions	RMSD	Interactions	RMSD
**Malic acid**	Ala 438, Arg 100, Arg 126, Arg 435 (H-bonding)	0.7528	Lys 97, Phe 103 (H-bonding)	2.6967	Lys 87 (H-bonding)	1.5581	Arg 179, Arg 182 (H-bonding), Arg 179 (cation- π interaction)	1.4156
**Dihydroxybenzoic acid**	Ala 438, Arg 100, Arg 126, Arg 435, Trp 122 (H-bonding)	1.8565	Arg 38 (H-bonding)	0.7020	Lys 87 (H-bonding)	1.5170	Arg 179, Arg 182 (H-bonding), Arg 179 (cation- π interaction)	2.9248
**Coumaric acid**	Ala 438, Arg 100, Arg 126, Arg 435, Trp 122 (H-bonding)	1.3974	Arg 120 (H-bonding)	1.2355	Lys 87 (H-bonding)	1.7174	Arg 30 (H-bonding)	2.4270
**Gallic acid**	Ile 115, Val 436 (H-bonding)	0.7528	Pro 95 (H-bonding)	0.8833	-	2.8237	Arg 179, Arg 182 (H-bonding)	1.4889
**Caffeic acid**	Ala 438, Arg 100, Arg 126, Arg 435, Trp 122 (H-bonding)	0.9062	Lys 64, Pro 65 (H-bonding)	1.2318	Ser 85 (H-bonding)	1.8186	Arg 30 (H-bonding)	0.8615
**Quinic acid**	Arg 100, Arg 126, Arg 435 (H-bonding)	1.702	Lys 9, Leu 96, Thr 10 (H-bonding)	2.3026	Lys 87 (H-bonding)	1.0609	Arg 179, Arg 182 (H-bonding)	2.9166
**Citric acid**	Ala 438, Arg 100, Arg 126, Arg 435, Trp 122 (H-bonding), Arg 100, Arg 435 (Ionic bond)	1.2329	Lys 97, Phe 103 (H-bonding)	2.9496	Lys 87 (H-bonding), Lys 87 (Ionic bond)	2.3101	Arg 179, Arg 182 (H-bonding), Arg 179 (Ionic bond)	2.2124
**Glucuronic acid**	Arg 100, Arg 126, Arg 435, Trp 122 (H-bonding), Arg 100, Arg 126 (Ionic bond)	0.7063	Lys 9, Gln 13 (H-bonding)	2.1881	Lys 87 (H-bonding)	1.3378	Arg 179, Arg 182 (H-bonding)	2.2640
**Kaempferol**	Arg 100, Cys 437 (H-bonding)	1.4216	Asn 88 (bonding)	1.8150	Lys 87 (H-bonding)	1.9803	Arg 179 (H-bonding)	2.6971
**Diosmetin**	Ala 438, Cys 437, Thr 303 (H-bonding)	2.0372	Pro 47 (bonding)	2.4411	-	2.73	Gln 175 (H-bonding)	2.5186

**Quercetin**	Cys 437, Thr 303 (H-bonding)	0.9062	Arg 120 (bonding)	2.7075	Leu 133 (H-bonding)	1.6192	Arg 30 (Solvent contact)	2.3451
**Protocatechuic acid**	Arg 100, Arg 126, Arg 435, Cys 437, Trp 122 (H-bonding)	1.2509	Lys 9, Glu 95 (H-bonding)	2.7230	Lys 87 (H-bonding)	2.2443	Arg 179, Arg 182, Gln 175 (H-bonding)	2.7200
**Isorhamnetin**	Ala 438 (H-bonding)	1.4845	Glu 116 (H-bonding)	1.8992	-	1.5759	Arg 179 (H-bonding)	2.0505
**Coumaric acid glucoside**	Arg 100, Arg 126, Arg 435, Trp 122, Thr 303, Cys 437, Gly 439 (H-bonding)	1.0350	Lys 64 (H-bonding)	2.9236	Lys 87 (H-bonding)	1.2000	Arg 179, Arg 182 (H-bonding)	1.7046
**Coumaroylquinic acid**	Ala 438, Arg 100, Arg 126, Arg 435, Trp 122, Pro 429 (H-bonding)	1.6765	Lys 43, Thr 111 (H-bonding)	1.5814	Lys 87 (H-bonding)	2.5392	Arg 179, Arg 182 (H-bonding), Asp 43 (Solvent contact)	2.0318
**Caffeic acid glucoside**	Ala 438, Arg 100, Arg 126, Arg 435, Trp 122 (H-bonding)	1.5466	Lys 64 (H-bonding)	2.4686	Ser 85, Lys 87 (H-bonding)	1.4528	Arg 179, Arg 182, Gln 175 (H-bonding)	1.4306
**Caffeoylquinic acid**	Arg 435, Ile 114 (H-bonding)	1.5928	Lys 48 (H-bonding)	1.7428	Lys 87 (H-bonding)	2.5341	Arg 179, Arg 182 (H-bonding)	1.5370
**Ferulic acid glucoside**	Thr 307, Pro 429 (H-bonding)	1.3020	Asp 109 (H-bonding)	2.3112	Leu 233 (H-bonding)	2.4020	Arg 179, Arg 182 (H-bonding)	2.8252
**Feruloylquinic acid**	Ala 438, Arg 100, Arg 126, Arg 435, Trp 122 (H-bonding), Arg 435 (Ionic bond)	1.9765	Glu 116, Arg 120, Lys 48 (H-bonding), Arg 120 (Ionic bond)	2.1286	Lys 87, Leu 233 (H-bonding)	2.6366	Arg 179, Arg 182 (H-bonding)	2.5210
**Diosmetin glucoside**	Thr 304, Cys 437, Gly 439, Pro 429 (H-bonding)	1.0311	Asn 88, Glu 95, Lys 9 (H-bonding)	1.9581	Leu 233 (H-bonding)	2.4748	Arg 179 (H-bonding)	2.2496
**Quercetin glucoside**	Arg 126, Arg 435, Cys 437, Ile 115, Trp 122 (H-bonding)	1.2376	Gln 22 (H-bonding)	1.9344	Lys 87, Leu 233 (H-bonding)	1.6565	Arg 179, Arg 182 (H-bonding)	1.5964
**Isorhamnetin glucoside**	Arg 435, Ala 438, Thr 303 (H-bonding)	1.2576	Asn 88, Glu 95 (H-bonding)	1.2269	Leu 233 (H-bonding)	1.6309	Arg 30 (H-bonding)	2.5610
**Silymarin®**	Arg 126, Gln 356 (H-	0.9760	Glu 52 (H-bonding)	1.3630	Leu 233 (H-	1.4592	Arg 30 (H-bonding)	2.1010

	bonding)				bonding)			
**Co-crystallized ligand (4-Methylpyrazole)**	Gly 300 (H-bonding)	1.4167	–	-	–		–	-

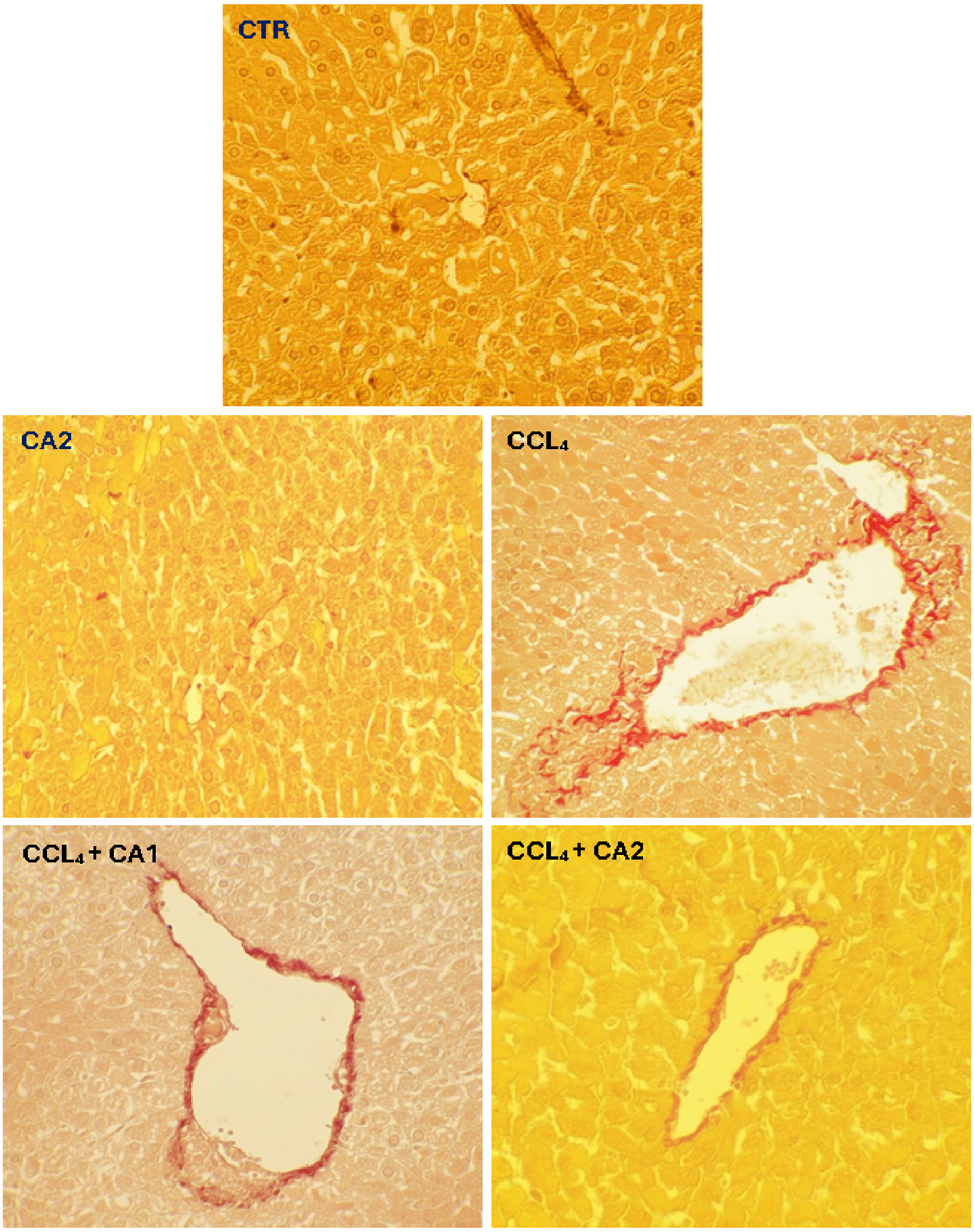
CTR
CA2
CCL_4
CCL_4 + CA1
CCL_4 + CA2

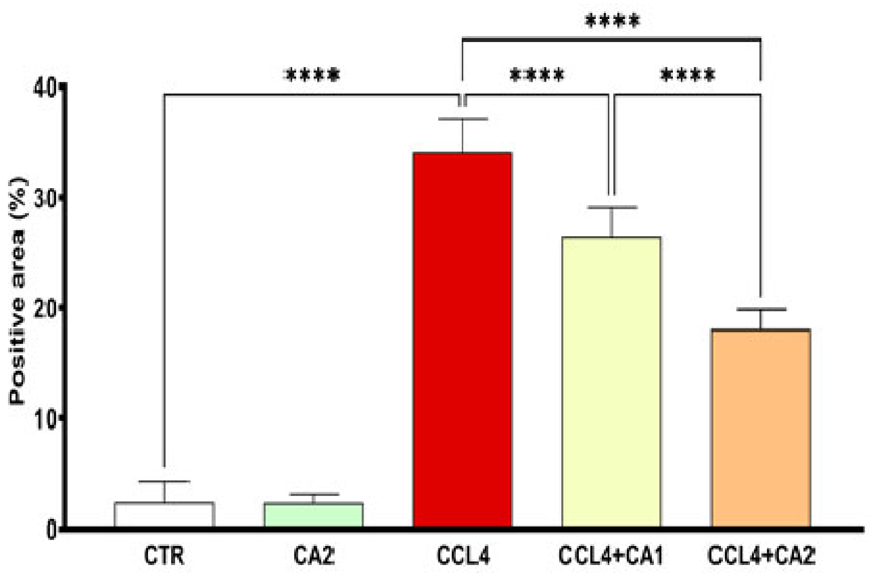

Figure S1. Representative photomicrographs of liver sections stained with Sirius Red showing collagen fibers (red) in different experimental groups. The red-stained areas correspond to collagen deposition and fibrotic changes, which are considered histopathological lesions indicative of chronic liver injury. Semi-quantitative morphometric analysis of collagen-positive areas was performed using ImageJ, and the results are presented in the accompanying bar graph.
